# Strategies for Rapid Muscle Fatigue Reduction during FES Exercise in Individuals with Spinal Cord Injury: A Systematic Review

**DOI:** 10.1371/journal.pone.0149024

**Published:** 2016-02-09

**Authors:** Morufu Olusola Ibitoye, Nur Azah Hamzaid, Nazirah Hasnan, Ahmad Khairi Abdul Wahab, Glen M. Davis

**Affiliations:** 1 Department of Biomedical Engineering, Faculty of Engineering, University of Malaya, Kuala Lumpur, Malaysia; 2 Department of Biomedical Engineering, Faculty of Engineering and Technology, University of Ilorin, Ilorin, Nigeria; 3 Department of Rehabilitation Medicine, Faculty of Medicine, University of Malaya, Kuala Lumpur, Malaysia; 4 Clinical Exercise and Rehabilitation Unit, Discipline of Exercise and Sport Sciences, Faculty of Health Sciences, The University of Sydney, Sydney, Australia; Universidad Europea de Madrid, SPAIN

## Abstract

**Background:**

Rapid muscle fatigue during functional electrical stimulation (FES)-evoked muscle contractions in individuals with spinal cord injury (SCI) is a significant limitation to attaining health benefits of FES-exercise. Delaying the onset of muscle fatigue is often cited as an important goal linked to FES clinical efficacy. Although the basic concept of fatigue-resistance has a long history, recent advances in biomedical engineering, physiotherapy and clinical exercise science have achieved improved clinical benefits, especially for reducing muscle fatigue during FES-exercise. This review evaluated the methodological quality of strategies underlying muscle fatigue-resistance that have been used to optimize FES therapeutic approaches. The review also sought to synthesize the effectiveness of these strategies for persons with SCI in order to establish their functional impacts and clinical relevance.

**Methods:**

Published scientific literature pertaining to the reduction of FES-induced muscle fatigue was identified through searches of the following databases: Science Direct, Medline, IEEE Xplore, SpringerLink, PubMed and Nature, from the earliest returned record until June 2015. Titles and abstracts were screened to obtain 35 studies that met the inclusion criteria for this systematic review.

**Results:**

Following the evaluation of methodological quality (mean (SD), 50 (6) %) of the reviewed studies using the Downs and Black scale, the largest treatment effects reported to reduce muscle fatigue mainly investigated isometric contractions of limited functional and clinical relevance (n = 28). Some investigations (n = 13) lacked randomisation, while others were characterised by small sample sizes with low statistical power. Nevertheless, the clinical significance of emerging trends to improve fatigue-resistance during FES included (i) optimizing electrode positioning, (ii) fine-tuning of stimulation patterns and other FES parameters, (iii) adjustments to the mode and frequency of exercise training, and (iv) biofeedback-assisted FES-exercise to promote selective recruitment of fatigue-resistant motor units.

**Conclusion:**

Although the need for further in-depth clinical trials (especially RCTs) was clearly warranted to establish external validity of outcomes, current evidence was sufficient to support the validity of certain techniques for rapid fatigue-reduction in order to promote FES therapy as an integral part of SCI rehabilitation. It is anticipated that this information will be valuable to clinicians and other allied health professionals administering FES as a treatment option in rehabilitation and aid the development of effective rehabilitation interventions.

## Introduction

Muscle atrophy, and the consequent alteration of the proportion of slow-twitch type I motor units (MU) to fast-fatigable type IIB (with low aerobic-oxidative enzymatic capacity) MU, are negative neuromuscular sequelae secondary to spinal cord injury (SCI) [1, 2]. As a consequence of these morphological and histochemical adaptations, resistance to rapid fatigue is impaired in denervated skeletal muscles compromised by upper-motor neuron lesions in the spinal cord [3]. Accordingly, the power output and exercise capacity of such muscles are diminished due to inactivity and unloading concomitant with post-SCI wheelchair confinement [4]. This is clearly evident in the decline of force-generating capacity of muscle (i.e. specific tension (N·cm^-2^)) [5], due to the problem of muscle fatigue in individuals with neurological disorders [6]. Current efforts to offset the issue have limited impact, due partly to the limitation of pathophysiologic understanding of muscle fatigue and its complications in this population [6, 7]. Consequently, there is on-going research interest into developing effective strategies to counteract the effects of rapid muscle fatigue, particularly during neuromuscular electrical stimulation for therapeutic and functional interventions in persons with neurological impairment, specifically SCI [8].

Neuromuscular electrical stimulation (NMES) applied over the human neuromusculature produces muscle contractions by depolarizing motor axons beneath the stimulating electrodes [9]. The generated muscle contractions result in therapeutic and/or functional gains by exploiting the adaptive potential of skeletal muscles fibres to increase the loading effect on joints. When evoking functional or performance gains, NMES has often been characterized as “functional electrical stimulation” (FES) [9]. FES has been applied to maintain, improve or restore muscle trophism, improve health and augment functional outcomes after SCI [9], in post-acute care, rehabilitation settings and exercise [10–12]. The usefulness of FES therapy to promote the ‘restoration’ of purposeful function has been demonstrated in several studies [11, 13, 14]. Yet, the inherent non-physiological response of paretic or paralyzed muscles (due to changes in their histological composition [2] and the reversal of usual MU recruitment order [15]) to electrical stimulation often leads to non-optimal recruitment of fast fatigable muscle fibres over fatigue-resistant fibres [16, 17]. Similarly, majority of evidence supports that the ordering of FES-induced MU recruitment is non-selective [15, 18], the consequent of which is the exaggerated metabolic cost of an electrically-evoked contractions that lead to rapid muscle fatigue [9, 18]. This limits the duration of functional tasks that FES may evoke [19]. Therefore, the need for practical solutions to the “rapid fatigue problem” is of paramount importance if FES therapy is to become more widespread in deployment for patients’ rehabilitation.

The low ‘take-up’ of FES interventions within conventional clinical practice, should not be assumed to indicate any lack of medical benefits, since scientific understanding of electrical stimulus-induced fatigue in the clinical population is still rudimentary [6, 20]. Previous studies, that have sought to improve fatigue-resistance during FES therapy, have investigated the effects of size of stimulating electrodes or their position over some specific locations (such as anatomical landmarks and motor points) [16], modulation of neuromuscular stimulation parameters [21], optimization of the mode and frequency of exercise [22] and biofeedback-controlled stimulation [23]. Although these prior studies were undertaken to improve the effectiveness of FES rehabilitation within clinical populations, none has consistently improved the fatigue resistance characteristics of paralyzed muscles [15]. Moreover, these techniques have not been fully incorporated into the routine clinical practice. Rather, the techniques have been more investigated for optimizing FES-assisted muscle contractions in able-bodied (AB) compared to SCI populations [21]. However, evidence supporting the successful transfer of the FES techniques from AB to clinical population remains poorly documented.

The current review sought to synthesize knowledge about effectiveness of fatigue-reduction strategies for persons with SCI in order to establish their functional impact and clinical relevance. Earlier, there have been some historical reviews on the implications and management of muscle fatigue in clinical populations, such as Binder-Macleod and Snyder-Mackler [24] and Maffiuletti et al., [25]. Apart from the populations considered by the authors of those study (*i*.*e*., AB and persons with SCI), only the manipulation of neuromuscular stimulus parameters was considered to improve FES contractions [24]. Subsequently, changes in the skeletal muscle characteristics following SCI have been highlighted in relation to the effects of fatigue resistance during FES therapy [26, 27]. Recently, Maffiuletti and colleagues [25] suggested a new strategy for FES deployment (*i*.*e*., multi current pathway FES). Although the authors’ paradigm produced more forceful muscle contractions, the rapid-fatigue cycle did not show any significant improvements compared with traditional FES strategies. Clearly, in the clinical environment, more widely accepted and ‘FES-efficient’ strategies need to be devised, and a comprehensive insight into an effective management of rapid muscle fatigue is needed.

## Methods

### Search strategy

Initially, 1933 articles within the electronic databases of Science Direct, Medline, IEEE Xplore, SpringerLink, PubMed, and Nature, and 2 additional articles identified through other sources (online request) were obtained from the earliest returned record until June 2015. The relevant search terms included ‘spinal cord injury’, ‘paralysis’, ‘paraplegia’, ‘tetraplegia’ with ‘muscle fatigue’, ‘reduction’, ‘delay’, ‘functional electrical stimulation’, ‘electrical stimulation’, therapy’, ‘contractions’, ‘walking’, ‘standing’ and ‘cycling’. The synonyms for the terms (e.g., ‘gait’ or ‘stepping’ for walking) were also included on the list of search terms. Further, we conducted a free search in Google Scholar using the reference listed in the primary citations in order to accommodate a wider context. Electrode databases were searched online through the University of Malaya, Malaysia library. However, the literature search was restricted to the English language only.

### Eligibility criteria

The eligibility criteria were set to accommodate the focus of the review which sought to evaluate the methodological quality as well as synthesize the effectiveness of muscle fatigue-reduction strategies during FES exercise for functional and therapeutic benefits in persons with SCI. The titles and abstracts of all identified studies were screened to determine eligibility. In the event that the title or abstract did not provide adequate information, such article was retrieved for full review. In a second round of filtering, the full text of all potentially relevant studies were reviewed against the following inclusion criteria by two reviewers (MOI and NAH): (i) the study participants were humans with SCI; (ii) the study objective involved the delayed onset of muscle fatigue *i*.*e*., assessed the ability of muscle to sustain a significant longer duration of repeated contractions following the administration of a specific strategy; (iii) at least one outcome quantified the improvement/or otherwise of resistance of rapid muscle fatigue in terms of endurance, force output (or its derivatives), number of contractions and contractile speed; (iv) study outcomes were measured based on a well-defined muscle fatigue reducing strategy. Studies with unclear protocol or data presentation were excluded. Four major categories of strategies were clearly evident *i*.*e*. optimization of electrode positioning, fine-tuning of stimulation patterns and other FES parameters, adjustments to the mode and frequency of exercise training, and biofeedback-assisted FES-exercise.

### Data extraction

In order to reduce the risk of bias, two reviewers (MOI and NAH) were independently involved in extraction of data for all outcomes derived from the four identified fatigue resistance strategies. Other reviewers (AKA and NH) verified the validity of the data before a final compilation, while two others (NH and GMD) checked the technical soundness, clarity and the flow of the study. Information on participants’ physical characteristics, study objectives, methodologies, interventions, outcomes/results and limitations, and future research suggestions were retained.

### Assessment of study quality

The quality of the studies was measured using the Downs and Black (D&B) Scale [28] relevant in assessing both randomised and non-randomised studies and adjudged a valid scale for methodological quality assessments [29]. The quality of each study was independently assessed by two reviewers using the D&B Scale [28]. A third “judge” was invited when the two reviewers disagree to settle the discrepancies. In line with other previously published reviews [30, 31] some criteria on the D&B scale (*i*.*e*., 17, 22 and 27) were considered inapplicable to the objective of the current review and, therefore, not graded. Thereafter, we noted significant variations between the levels of spinal lesion of the participants. In general, subjects with complete and incomplete lesions, as well as those with acute and chronic injuries were grouped together. A meta-analysis could not be conducted due to significant variations in the methods used to evaluate the outcomes, subject heterogeneity and diverse experimental methodologies. The maximum score on the D&B scale was normalised to 100% as some criteria were relevant to some studies and irrelevant to others.

## Results

### Included studies

**Fig 1:**
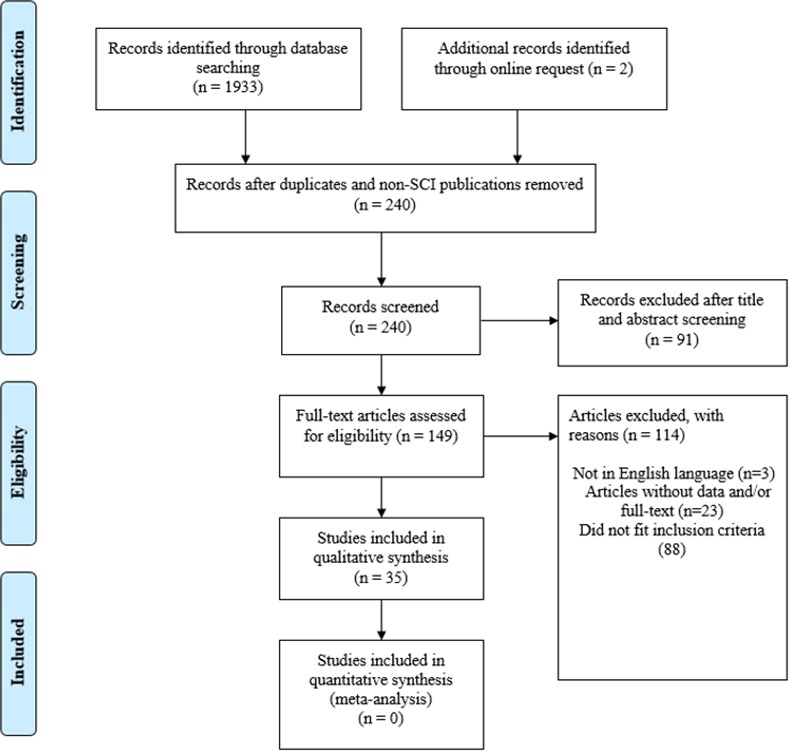
PRISMA flow chart for included and excluded studies in the systematic review on fatigue reduction strategies during FES exercise.

At the outset, 1935 citations were generated and 1900 failed to fit the inclusion criteria. Thirty-five studies met the inclusion criteria and were critically analysed for this review (Fig 1). Out of these studies, eighteen assessed the modification of neuromuscular stimulation patterns, five investigated the optimization of electrode position, ten evaluated the optimization of exercise interventions and two appraised the ‘quality’ of exercise when augmented by biofeedback-controlled FES muscle contractions. Outcome measures were assessed based on the type and mode of muscle contractions (*i*.*e*., isometric versus non-isometric- isotonic/isokinetic). The characteristics of the included studies are presented in Tables 1–4.

**Table 1:** Overview of included studies: Modification of stimulation patterns.

Study	D&B Quality Rating	Population Age, TSI: years (Mean± SD)	Methods	Functional Implications
Karu et al., 1995 [35]	46%	**Participants:** 4(C5/6-T10) SCI and 27 AB, **Age:** SCI: 34.5±9, AB: 19–38, **Muscle(s):** Quadriceps, **TSI:** SCI: 10.8±10.4	**Contraction:** Isometric. **Objective:** (i) to investigate if nonlinear summation due to closely spaced stimulation pulses applies to human skeletal muscle; (ii) to identify the *N*-let pulse parameter that will produce the most force per pulse; (iii) to verify the hypothesis that optimal *N*-let trains will enable significantly longer FES muscle contractions. **Study design:** The quadriceps muscle was stimulated for 1 hour each day and exercised on a FES-aided bicycle 3 times a week. The optimal pulse interval was calculated by parameters of *N*-let pulse trains for number of pulses (*N)* = 1–6 in order to obtain the most force per pulse. The duration of torque tracking was used to compare the different modes of stimulation. **Stimulation pattern:** *N*-let pulse trains (*i*.*e*., a set of closely spaced stimulation pulses), PW: 300 μs, Frequency: 40 Hz.	Optimal *N*-let stimulation produced 51% longer isometric torque tracking compared to the traditional singlet stimulation in SCI participants.
Graupe et al, 2000 [45]	38%	**Participants:** 1T7 SCI, **Age:** NM, **Muscle(s):** Quadriceps, **TSI:** NM	**Contraction:** Non-isometric. **Objective:** Preliminary report on the offset of rapid fatigue during FES standing and stepping by randomly modulating inter-pulse interval. **Study design:** Several digital signal processing chip were processed, each with different modulation parameters (*i*.*e*., in 24 pulses/s, with inter-pulse interval of 42 ms and stochastic modulation rates of 0 ms fixed rate, ±5 ms, ±10 ms, and ±20 ms. The maximum stimulation was applied to the participant’s quadriceps while seated in a fixed position relative to the chair. The time at which the leg touched the floor was recorded during horizontal leg extension. **Stimulation pattern:** 10% (±5 ms) at 42 ms inter-pulse interval (24 pulses/s).	The leg extension time was significantly extended by 36.63% longer during 24 pulses/s stimulation than with the same pulse interval (42ms) with no stochastic modulation.
Griffin et al., 2002 [36]	46%	**Participants:** 6(C5-C6) SCI and 6 AB, **Age:** SCI: 33±5, AB:37±3 **Muscle(s):** Thenar, **TSI:**10.3±3	**Contraction:** Isometric. **Objective:** To determine the pattern of pulses that elicited maximal force from paralyzed and control thenar muscles. **Study design:** The distal and proximal electrodes were placed over the metacarpophalangeal joint and the base of the thumb, respectively. A ground wire was placed along the wrist crease. The thumb was extended and strapped against a force transducer that registered both abduction and flexion forces at right angles to each other. Resultant force was calculated off-line. **Stimulation pattern:** Doublet pattern (*i*.*e*., a brief inter-pulse interval) and twitch contractions. PW: 50 μs was used to determine the best site for stimulation. Frequency: 50 Hz.	In paralyzed and control muscles, the maximal force and force-time integral were produced when brief (5–15 ms) interpulse interval was followed by longer intervals.
Godfrey et al., 2002 [34]	50%	**Participants:** 8(C4-C6) SCI, but analyzed the results of 4, **Age:** 29–44, **Muscle(s):** Thenar, **TSI:** 4–6	**Contraction:** Isometric. **Objective:** To compare the fatigability of paralyzed thenar muscles when the median nerve was stimulated supramaximally (SupMax) and submaximally (SubMax).**Study design:** The two patterns of 2 min intermittent fatigue stimulation were performed on each participant separated by 2 days. The stimuli resulted in activation of part ‘SubMax’ and all ‘SupMax’ of the thenar muscles. **Stimulation:** SubMax and SupMax. PW: 300 μs, Frequency: 40 Hz, Amplitude: Variable, increment in 0.1 mA steps based on maxima and supramaximal M-wave recordings.	The relative force loss: 19±3% and 48±6%, the decline in FTi: 50±12% and 69±3% at 105 sec., and slowing of half relaxation time: (108±30.6 ms to 465.5±126.4 ms) for supmax as against submax (83±9 ms to 235.4±54.4 ms), were always significantly (*P*<0.001) less when only submaximally rather than supramaximally stimulated muscles were fatigued. Submaximal stimulation may reduce muscle fatigue because it recruits more fatigue-resistant motor units.
Eser et al., 2003 [42]	55%	**Participants:** 19 (C5-T10) SCI, **Age:** 31.9±12.3, **Muscle(s):** Hamstring, Gluteal, and Quadriceps, **TSI:** 6.4±3	**Contraction:** Non-isometric. **Objective:** To investigate if stimulation frequencies higher than 30 Hz would increase the PO of FES cycling for 30 min duration in trained SCI patients, and if higher stimulation frequencies might produce more fatigue as measured by the PO produced in the last minute of each session. **Study design:** In each week, sessions were scheduled for random order frequencies of 30, 50, and 60 Hz at cycling cadence range of 40–50 rpm. If the speed exceeded 50 rpm, the controller increased the resistance by one step; and if it fell below 40 rpm, it decreased the resistance by one step. Stimulation frequency was set at 30 Hz until a training duration of 30 min was reached, then the frequency was varied randomly from session to session. Only training sessions of 30 min were included in the data analysis. **Stimulation pattern:** Frequency: 30, 50, and 60 Hz PW: 300 μs for 18 participants and 400 μs for one participant (due to the thicker subcutaneous fat). Amplitude: 140 mA (Preset maximum amplitude).	Mean power output production between 30 and 50 Hz, as well as between 30 and 60 Hz (*P*<0.001) were significant, while difference between 50 and 60 Hz (*P* = 0.57) was insignificant.
Thomas et al., 2003 [38]	46%	**Participants:** 10 (C4-C6) SCI and 5 AB, **Age:** SCI: 5±1 AB: 28±2, **Muscle(s):** Thenar, **TSI:** 10±1	**Contraction:** Isometric. **Objective:** To investigate whether variable versus constant frequency pulse trains could reduce the fatigability of paralyzed thenar muscle. **Study design**: Four experiments, separated by one day, were performed on each participant. A train of supra-maximal stimulation of 50 Hz for 1 s was delivered to check consistency of setup between experiments. **Stimulation pattern:** Supramaximal stimuli were delivered in the following order:5 pulses at 1 Hz to evoke twitches.2 pulses separated by 5 ms, repeated 3 times at 1 s intervals to assess the doublets’ evoked force.50 Hz for 1 s to evoke maximal force.13 trains of stimuli, each 300 ms duration.	The force decline (to 40–60% initial fatigue force or to 29–36% initial 50 Hz force) was similar whether constant or variable pulse patterns were used at high or low frequencies.
Kebaetse et al., 2005 [12]	50%	**Participants:** 9(C5/6-T9/11) SCI, **Age:**17.1±4.5, **Muscle(s):** Quadriceps, **TSI:** 36.6±30.6	**Contraction:** Non-isometric. **Objective:** To determine the effects of both constant stimulation frequency and increasing the stimulation frequency across contractions on the ability of paralyzed muscles to perform repetitive, non-isometric contractions. **Study design:** Three protocols were each tested during separate sessions: 20 Hz trains of pulses followed by 66 Hz trains (C20+66), 33-Hz trains followed by 66 Hz trains (C33+66), and 66 Hz trains alone (C66). For each frequency, stimulation was repeated until the knee failed to produce a 50deg excursion. This approach allowed the evaluation of the response to stimulations with 20, 33, and 66 Hz and combinations of 20 and 66 Hz and 33 and 66 Hz trains. **Stimulation pattern:** (C20+66), (C33+66), 66, 33, 20 Hz. PW: 600 μs. Amplitude: Stimulation intensity (mA) to elicit a force equal to the maximum twitch force using a 1s long, 20 Hz train was used.	The C20 and C33 did not differ (mean, 41.0±12.6 and 42.0±12.3 excursions, respectively), and each produced more excursions than the C66 protocol. The C20+66 and C33+66 protocols produced 51.4±15.0 and 44.9±13.6 excursions, respectively, and the C20±66 was the best protocol overall, at *P*≤0.05.
Decker et al., 2010 [47]	55%	**Participants:** 12 (C3-T10) SCI, **Age:**37±3 **Muscle(s):** Quadriceps, **TSI:** 10.3±2.5	**Contraction:** Non-isometric. **Objective:** To compare paralyzed quadriceps FES-cycling performance during alternation and co-activation stimulation protocols. **Study design:** Each participant performed FES-cycling with two different quadriceps stimulation protocols in two different experimental sessions separated by 48 hours. During the alternation protocol, each of the muscle groups rested for two 100 ms “off” periods in each 400 ms burst. The standard ‘‘co-activation” protocol (SCP) consisted of a 400 ms constant frequency train delivered simultaneously to the superficial quadriceps muscles (VM, VL, and RF). The “alternation” stimulation protocol (ASP) consisted of a series of four 100 ms constant frequency trains where the VM and VL were stimulated 100 ms out of phase with the RF. Participants’ FES cycling performance limits and their muscle mechanomyographic signals (MMG) were compared based on the two stimulation protocols. **Stimulation pattern:** SCP *i*.*e*. intermittent simultaneous activation of the entire quadriceps muscle group for 400 ms.ASP *i*.*e*. alternately stimulating the RF for 100 ms and the VM and VL muscles for 100 ms, with two sets and a 400 ms burst.PW: 500 μs, Frequency: 50 Hz, Amplitude: up to 140 mA.	There was no significant difference (*P* = 0.073), between the average cycling cadences (28 rpm) in the two protocols. The ASP produced an average of 2.36 minimum ride time longer (*P* = 0.024), and 0.4 miles longer (*P* = 0.008) virtual distances travelled and used lower stimulation intensity levels with no differences in average mechanomyogram (an indicator of the level of neuromuscular contractions) amplitudes compared to the SCP.
del-Ama et al., 2012 [43]	50%	**Participants:** 6(C4-D7) SCI, **Age:** 51±15, **Muscle(s):** Knee Flexor and extensor, **TSI**:NM	**Contraction:** Isometric. **Objective:** To investigate muscle fatigue responses of two customized FES protocols and a control (FM- Frequency modulation, AM- Pulse amplitude modulation; and CFA-Constant frequency and amplitude). **Study design:** A Fatigue protocol of the knee flexor and extensor was conducted in order to compare the strategies including stepwise decrease of FM, AM and the control strategy (CFA). **Stimulation pattern:** FM, AM and CFA, PW: 350 μs, Frequency: 70 Hz, Amplitude: 90% of tolerable, Train: 14 s and 43% duty circle.	FM provides higher normalized force-time integral (FTi) than AM (*P* = 0.075), and CFA (*P* = 0.225).Flexor showed less force and lower fatigue than extensor muscle.FM may optimize toe clearance during swing phase of stepping.
Chang and Shields, 2011 [37]	42%	**Participants:** 14(11 chronic and 3 acute, at T6 and above) SCI, **Age:** 32±6 and 28±4 for chronic and acute SCI respectively, **Muscle(s):** Soleus, **TSI:** ≥ 3 for chronic SCI and < 2 weeks for acute SCI	**Contraction:** Isometric. **Objective:** To examine if the doublet train activation in paralyzed muscles will enhance the magnitude of torque after fatigue and potentiation. **Study design**: The protocol involved an assessment of force elicited by a constant frequency train of stimuli (CT) or by a constant frequency train preceded by a doublet with a 6 ms inter-pulse interval (DT). With the knee flexed to 90deg, the soleus muscle produces the majority of plantar flexion torque. After initial activation with CT or DT, participants received a repetitive activation to fatigue the soleus. Then, the CT or DT protocol was repeated at 5, 10, 15, 20 min after the caseation of the fatigue bout. Participants performed the protocols on 2 occasions, with 10 days rest interval. **Stimulation pattern:** CT and DT, Contractions: 120 with 12 pulses train. Duty circle: 330 ms on and 670 ms off, Frequency: 20 Hz, Amplitude: Range of 125–175 mA.	The increments in nPT at 15 and 20 min with the DT protocol (*i*.*e*., 23% and 29%) were greater than the increments at 5 and 10 min (*i*.*e*., 10% and 12%) *P* <0.05. Therefore, the DT protocol, as compared with the CT protocol, was more effective during low-frequency fatigue as time after fatigue increased.
Graham et al., 2006 [46]	50%	**Participants:** 7(C6/C7-T8) SCI, **Age:**31.2±6.2, **Muscle(s):** quadriceps and tibialis anterior, **TSI:** 0.25 to 13 for chronic SCI and < 2 week for acute SCI	**Contraction:** Isometric. **Objective:** To investigate if FES induced muscle fatigue could be reduced by random modulation of FES parameters. **Study design:** FES was applied to the quadriceps and tibialis anterior muscles bilaterally using surface electrodes. In random trials, a method of stochastic modulation of the stimulation parameters involving four modes of stimulation (*i*.*e*., constant stimulation (CF), randomised frequency (RNF), randomised current amplitude (RC) and randomised PW) was used. It involved moderate (15%) variations updated every 100 ms and centered around 40 Hz. The isometric force was measured, and the time for the force to drop by 3 dB (fatigue time) and the FTi were determined. **Stimulation pattern:** CS, RNF, RC, randomised, PW: 250 μs, Amplitude: Range 34–110 mA, Frequency: 40 Hz	The difference between the fatigue time measurements for the four modes of stimulation (*P* = 0.311), and between the FTi measurements (*P* = 0.436) were insignificant.
Thrasher et al., 2005 [8]	55%	**Participants:** 7(C6/C7-T8) SCI, **Age:**31.2±6.2 **Muscle(s):** Quadriceps and tibialis anterior, **TSI:** 7.2±4.3	**Contraction:** Isometric. **Objective:** To test a hypothesis that FES-induced muscle fatigue could be reduced by randomly modulating the pulse frequency, amplitude, and pulse width in a range of ±15%. **Study design:** Four different randomly ordered patterns of FES were applied: constant stimulation, randomized frequency, randomized amplitude, and randomized pulse width. All the stimulation parameters were randomised: ±15%The time for the force to drop by 3 dB (fatigue time) was compared between trials. **Stimulation pattern:** PW: Variable, mean: 250 μs, Frequency: Variable, mean: 40 Hz, Amplitude: 34–110 mA *i*.*e*. varying with participants and muscle groups.	There was no significant difference between the fatigue-time measurements for the four modes of stimulation (*P* = 0.329) investigated.
Bickel et al., 2004 [39]	42%	**Participants:** 22(10 Acute (C3-C8) and 12 Chronic (C6-T8)) SCI and 10 AB, **Age:** Acute SCI: 28±2 Chronic SCI: 36.2±6AB: 16.2±4.1, **Muscle(s):** quadriceps, **TSI:** 23±5weeks for acute 8±2 years for chronic SCI	**Contraction:** Isometric. **Objective:** To test a hypothesis that variable frequency train (VFT) stimulation would augment torque-time integral in SCI population. **Study design:** The muscle was stimulated with constant frequency trains (CFTs) (six 200 s square wave pulses separated by 70 ms) or VFTs (a train identical to the CFT, except that the first 2 pulses were separated by 5 ms). **Stimulation pattern:** PW: Variable, Frequency: Variable	After 180 contractions (50% duty cycle), isometric PT decreased 44, 56, and 67%, in the AB, acute SCI and chronic SCI groups, respectively. In fatigued muscle, VFTs enhanced the TTi by 18% in AB participants and 6% in chronic SCI patients, and had no effect in acute SCI patients when compared to the corresponding CFT.
Scott et al., 2007 [21]	50%	**Participants:** 13 (Mid thoracic and cervical (n = 4) level) SCI, **Age:**16.2±4.1, **Muscle(s):** Quadriceps, **TSI:** 3.82±3.62	**Contraction:** Isometric. **Objective:** To investigate the FES-evoked force responses of paralyzed quadriceps muscles to 3 different stimulation patterns (CFT, VFT and DFT). **Study design:** Both non-fatigue and fatigue protocol were performed. 6-pulse CFTs, VFTs, and DFTs were delivered at mean frequencies of 10, 20, 33, 50, and 100 Hz. *Non-fatigue*: Stimulation trains delivered at a rate of 1 every 20 s to avoid fatigue. A sequence of fifteen 6-pulse testing trains (3 train types at 5 frequencies) delivered in a random order and then repeated in reverse order for a total of 30 non-fatigued testing trains. *Fatigue*: After a 5 min rest, 110, 13-pulse, 40-Hz CFTs delivered at a rate of 1 every second (300 ms on, 700 ms off, 30% duty cycle) was applied. The intensity for each participant was set and held unchanged for the rest of the sessions. **Stimulation pattern:** CFT, VFT and DFT, PW: 600 μs, Frequency: Variable, Amplitude: Depended on the stimulation pattern.	In the non-fatigued and fatigued conditions, the VFT and DFT peak forces were greater than the CFT peak forces at 10 Hz (*P* = 0.001). In the fatigued condition the 20 Hz VFT peak forces were greater than the CFT peak forces (*P* = 0.05), and there was a trend for the DFT peak forces to be greater than the CFT peak forces (*P* = 0.07) but no significant effect of train-type on the FTi(s).
Scott et al., 2005 [14]	50%	**Participants:** 9(7 Thoracic and 2 Cervical levels) C-SCI were recruited, however, only 6 participants’ data were analyzed, **Age:** 16.7±1.6, **Muscle(s):** Quadriceps, **TSI:** 0.83–8.25	**Contraction:** Isometric. **Objective:** To compare the number of muscle contractions that reached or exceeded a targeted isometric peak force when using CFTs followed by DFTs to the number of contractions produced when only CFTs or DFTs were used. **Study design:** Two test sessions were conducted separated by 24 hours. Maximum twitch force (MTF) testing was performed in random order and the force responses of paralyzed muscles to a series of single pulses (600 μs) were recorded. The target force used in the experiments was 75% of each participant’s MTF. **Stimulation pattern:** Combination of CFTs and DFTs, CFTs alone, and DFTs alone.PW: 600 μs, Amplitude: 51 mA for maximum SDSS and SES was 64 mA. Frequency: 40 Hz	The combination of CFTs followed by DFTs attained the targeted isometric force level (*P*<0.05), 14% more often than the CFTs alone and 18% more often than the DFTs alone. The switching train types (*i*.*e*., CFTs to DFTs) may be a useful strategy to subvert rapid muscle fatigue during sit-to-stand and stepping.
Chou et al., 2008 [41]	50%	**Participants:** 8(C7-T10/T11) SCI, **Age:**14.63±1.77**Muscle(s):** Quadriceps, **TSI:** 14.6±1.8	**Contraction:** Isometric. **Objective:** To compare the effectiveness of progressively increasing stimulation intensity (PISI), progressively increasing frequency (PIF), or progressively increasing both frequency and intensity (PIFI) on paralyzed quadriceps femoris muscle force maintenance during repetitive activation. **Study design:** Two testing sessions were conducted separated by 48 hours. Each session involved one of the two fatiguing protocols and the order of testing for each protocol was randomly assigned to each participant. The electrode connected to the anode of the stimulator was placed over the motor point of the RF, and the cathode was placed over the motor point of the VM transducer pad positioned 2.5cm proximal to the lateral malleolus. Each participant’s maximal twitch force was determined with a stimulation pulse of 600 μs delivered every 10 sec. Stimulation voltage was increased from 0 V to 135 V in 5 V increment until a plateau in the maximum peak twitch force occurred. **Stimulation patterns:** PISI, PIF and PIFIPW: 600 μs, Frequency: 60 Hz	PIFI contractions generated an average number of 189.88±53.33 contractions significantly (*P*<0.05) more than PIF followed by PISI (*i*.*e*., 122.75± 26.56). Regardless of the order, PIFI contractions were more successful than PISI (97 contractions) or PIF (62 contractions) alone.
Deley et al., 2015 [20]	55%	**Participants**: 10 (C-T12) SCI, **Age**: SCI: 33.9±1.2, **Muscle(s)**: Quadriceps, **TSI**: 9.5±2.5	**Contraction:** Isometric. **Objective:** To compare the responses of muscle torque and fatigue to CFT and VFT stimulation patterns during strength training exercise. **Study design:** Two test sessions were conducted separated by 24 hours. During each session, isometric muscle torque was measured under 2 sequential electrical stimulation train patterns (*i*.*e*., CFT and DFT).**Stimulation pattern: CFT:** 6 s of contractions was generated by a frequency of 40 Hz and PW of 450 μs, **VFT:** 6 s of contractions was generated by a frequency of 80 Hz doublet followed by a 20 Hz stimulation with 450 μs pulse width.	Target torque was achieved more times with VFT alone (VFT: 6.7±0.8 vs. CFT: 3.5±0.2 contractions, *P*<0.05) and when followed with CFT pattern (VFT-CFT: 10.3±1.2 vs. CFT-VFT: 6.9±1.2 contractions, *P*<0.05). VFT generated less fatigue contractions as compared to CFT.
Gorgey et al., 2014 [40]	70%	**Participants**: 10 (C5-T10) C-SCI, **Age**: 44±10 **Muscle(s)**: Quadriceps, **TSI**: > 1	**Contraction:** Isometric. **Objective:** To study the effects of using different pulse widths (200, 350, and 500 μs; P200, P350, and P500, respectively) on energy expenditure (EE) and muscle fatigability following FES cycling. **Study design:** Three randomly administered FES cycling protocols (P200, P350, P500) separated by 1 week, were conducted. Resting, exercise, and recovery heart rate (HR), blood pressure (BP), and EE were monitored across the three sessions. To obtain force-frequency curves, the fatigue tests were conducted before, immediately after, and at 48 to 72 hours after each cycling session with pulse with of 400 μs and 3s contraction time. Fatigue was assessed by measuring the torque elicited by quadriceps muscle group at 10, 20, 30, 40, 50, 60, 80, and 100 Hz (randomized) before and immediately after cycling. **Stimulation pattern:** P200, P350, and P500PW: 200, 350, and 500 μs for cycling; 400 μs for fatigue measurement. Pulse amplitude: 140 mA, 140 mA and 110 mA for knee extensor. Knee flexor and gluteus maximus muscle groups, respectively. Frequency: 33.3 Hz for cycling.	No significant (*P* = 70) effect of pulse width modulation on the muscle fatigability.

Abbreviation: SCI- Persons with spinal cord injury; AB- Able-bodied; M- mean; SD- Standard deviation; TSI-Time since injury; PW- Pulse width; CFT- Constant frequency train; VFT- Variable frequency train; DFT- Double frequency train; nPT-Normalized peak torque; TTi- Torque-time integral; NM-Not mentioned; SDSS- Spatially distributed sequential stimulation; SES- Single active electrode stimulation; RF- Rectus femoris; VM- Vastus medialis; VL- Vastus lateralis muscle; PO- Power output; PT- Peak torque.

**Table 2:** Overview of included studies: Optimization of electrode positioning.

Study	D&B Quality Rating	Population Age, TSI: years (Mean± SD)	Methods	Electrode	Functional implications
Popovic and Malesevic, 2009 [49]	41%	**Participants**: 6(C6/C7-T11/T12) complete paraplegia and 1 (TH9/TH10) incomplete tetraplegia, **Age**: 39±17, **Muscle(s)**: Quadriceps, **TSI**: 7.2±1.6 months	**Contraction:** Isometric. **Objective:** To verify if an asynchronous low frequency activation of pads within a multi-pad electrode positioned over the quadriceps muscle will result in a stronger fused contractions comparable to the force elicited with a single large electrode activated at a higher frequency. **Study design:** Protocol 1: The intensity of stimulation was adjusted to produce maximum joint torque. Protocol 2: At maximum joint torque, three series of measurements of knee joint torque resulting from a continuous electrical stimulation were obtained with two 5 min rest time. The interval between the start of stimulation and the instant when the joint torque declined below 70% of the maximum torque (*i*.*e*., fatigue interval) was also determined. **Stimulation pattern:** PW: 500 μs, Frequency: 30 Hz for HPF and 16 Hz for LPF, Amplitude: 88±37 mA	**Configuration:** HPF-Pals Platinum square- (7 × 10 cm) as the anode and cathode. LPF- Four oval cathodes (4 × 6 cm) and one square anode (7 × 10 cm). **Anatomical position:** HPF-Cathode electrode at the top and the anode at the distal part of the quadriceps, LPF- Anode at the distal while the four cathodes were distributed over the quadriceps such that no current pathway between the cathode and anode intersected.	The fatigue interval was extended more than 150% in the LPF (multiple-electrode) configuration as compared with HPF (two-electrode).
Malesevic et al., 2010 [50]	45%	**Participants**: 6 (C6/C7-T11/T12, complete paraplegia) and 1 (TH9/TH10) incomplete tetraplegia) **Age**: 38±12.6 **Muscle(s)**: Quadriceps, **TSI**: 7.2±1.6 months.	**Contraction:** Isometric. **Objective:** To investigate if daily FES with LPF protocol could train paralyzed muscles to become less fatigable as compared with HPF. **Study design:** The treatment included a 30 min daily session for 20 days. One leg was treated with the HPF protocol and the other with the LPF protocol. Knee-joint torque was measured before and after the therapy to assess the time interval before the knee-joint torque decreased to 70% of the initial value. **Stimulation pattern:** PW: 500 μs, Frequency: 30 Hz for HPF and 16 Hz for LPF, Maximum pulse amplitude:150 mA	**Configuration:** As detailed in reference [49]	The increase in the fatigue interval due to training were significant (*P*<0.05) for both protocols while the difference in the increase of the fatigue interval was not significant (*P*>0.05).HPF could be recommended for strength training, while LPF may be better suited for prolonged stimulation essential for stepping and grasping.
Nguyen et al., 2011 [51]	55%	**Participants**: 1(T3/T4) SCI, **Age**: NM, **Muscle(s)**: Triceps surae, **TSI**: 4	**Contraction:** Isometric. **Objective:** To investigate the fatigue reducing ability of SDSS pattern as compared to SES. **Study design:** *SDSS*: Applied by sending a stimulation pulse to each electrode one after another with 90deg phase shift between successive electrodes. *SES*: Applied by a single electrode. Each mode with resultant frequency of 40 Hz was applied for six days over twelve separate days. Isometric ankle torque was measured during fatiguing stimulations lasting 2 min. Fatigue measurements used included comparison: fatigue index (FI) (*i*.*e*., final torque normalized to maximum torque at the end of 2 min stimulation), fatigue time (FT) (*i*.*e*., time for torque to drop by 3 dB), and torque-time integral (TTi) (*i*.*e*., over the entire 2 min fatigue stimulation trial). **Stimulation pattern:** SDSS and SESPW: 250 μs, Amplitude: 51 mA for maximum SDSS and SES was 64 mA, Frequency: 40 Hz	**Configuration**: *SDSS-* Four separate (4.5 × 2.5 cm) were used as active electrode and a single (9 × 5 cm) was used as the reference. *SES*- Two separate (9 × 5 cm) were used as active and reference electrodes. **Anatomical position**: *Active electrode*- Located on the calf, near the gastrocnemius muscle at the distal part of the quadriceps *Reference*- Located above the achilles tendon.	SDSS produced 234% higher torques (compared with SES) at the end of the 2 min stimulation relative to the maximum torque with an improvement of 171% torque time integral (*P*<0.001).
Sayenko et al., 2014 [52]	50%	**Participants**: 17(C5-T12) SCI and 11 AB, **Age**: SCI: 44.6± 12AB: 25.7± 5.6, **Muscle(s)**: knee extensors, flexors, planter flexors and dorsiflexors, **TSI**: 8±8	**Contraction:** Isometric. **Objective:** To explore the fatigue-reducing ability of SDSS for some lower limb muscle groups in the healthy volunteers and persons with SCI. **Study design**: SDSS was delivered through 4 active electrodes applied to the 4 muscle groups investigated, sending a stimulation pulse to each electrode one after the other with 90deg phase shift between successive electrodes. To indicate the decay of muscle force during the fatiguing stimulation, the fatigue index (FI) was defined as the torque at the end of the 2 min stimulation normalized to the maximum torque. The SDSS and SES protocol were conducted in different days with 1-day rest in between. **Stimulation pattern:** SDSS and SESA bout consisting of 120 trains was used, each composed of 12 pulses and spaced 1 s apart, resulting in 120 muscle contractions. Resultant frequency: 40 Hz, PW: 300 μs	**Configuration:** *SDSS-* Four separate (3 of (4.5 × 2.5 cm) and 1 of (2.5 × 2.5 cm; for dorsiflexors)) were used as active electrode. *SES-* A pair of (9 × 5cm) were used as active and reference electrodes except for dorsiflexors on which (5 × 5cm) electrodes were used. **Anatomical position:** NM	Fatigue index of SDSS was greater than that of SES as follows: Knee extensor: (*P* = 0.004)Knee flexors: (*P*< 0.001)Planter flexors: (*P*< 0.001)Dorsiflexors: (*P* = 0.005)Torque peak mean of SDSS was greater than that of SES: Knee extensor: (*P* = 0.006)Knee flexors: (*P*< 0.001)Planter flexors: (*P*< 0.001)Dorsiflexors: (*P* = 0.004).
Downey et al., 2014 [33]	54%	**Participants**: 4(C5-T11) SCI and 4 AB, **Age**: SCI: 47.25± 14AB: 20–27, **Muscle(s)**: Quadriceps, **TSI**: 7.32±4.7	**Contraction:** Isometric. **Objective:** To compare the fatigue characteristics of high- and low-frequency asynchronous stimulation (AS) as well as high- and low-frequency conventional stimulation (CS). **Study design:** Four protocols were examined: 8 Hz asynchronous stimulation (A8), 16 Hz asynchronous stimulation (A16), 32 Hz conventional stimulation (C32), and 64 Hz conventional stimulation (C64). During AS, each channel utilized the same current amplitude, but the stimulation pulses were interleaved across the stimulation channels (*i*.*e*., AS of 16 Hz with four channels resulted in a composite stimulation frequency of 64 Hz). **Stimulation pattern:** PW: 350 μs, Frequency: 16, 32, and 64 Hz, Amplitude: 126 mA	**Configuration and position:** CS: a single stimulation channel with a pair of 3” x 5” Valutrode® surface electrodes placed over the quadriceps femoris muscle group.AS: four channels of stimulation utilizing four electrodes placed distally (1.5” x 3.5” Valutrode®) and two electrodes placed proximally (2” x 3.5” Valutrode®).	Mean fatigue time of A8 was significantly (F = 22:33; *P* = 1.379E-7) longer than that of A16, C32, and C64;A16 was significantly longer than C32 and C64; and C32 was significantly longer than C64.

Abbreviations: SCI- Persons with spinal cord injury; AB- Able-bodied; HPF- High pulse frequency stimulation; LPF- Low pulse frequency stimulation; TSI: Time since injury; PT-Peak torque; TTi-Torque-time integral; SDSS- spatially distributed sequential stimulation; SES- single active electrode stimulation.

**Table 3:** Overview of included studies: Optimization of exercise training.

Study	D&B Quality Rating	Population Age, TSI: years (Mean± SD)	Methods	Duration	Functional implications
Shields and Dudley-Javoroski, 2006 [22]	48%	**Participants**: 7(C5-T10) SCI, **Age**: 29.14±8.40, **Muscle(s)**: Flexor muscles, **TSI**: Within 6 wks.	**Contraction:** Isometric. **Objective:** To determine the long-time effect of FES long time training effect on PT, fatigue index, TTi and contractile speed. **Study design**: Within subject control design. Supramaximal stimulation of 10-pulse train (15 Hz; duration: 667 ms) every 2 s was used. Participants completed 4 bouts of exercise during each session (A bout of exercise consisted of 125 trains). Implication on the distal tibia trabecular BMD was observed. **Stimulation pattern:** PW: 667 ms, Frequency: 15 Hz, Amplitude: 0–200 mA	2 to 3 years. Laboratory based test and 2 to 4 times monthly,4 stimulation bouts per day on 5 days each week with 5 min of rest between each bout.	At 2 years, the untrained limbs showed a decrease (*P* = 0.05) in peak torque while the trained limb generated higher torque, ability to sustain work (TTi) and fatigue resistance. The BMD was, on average, 40 mg/cm3 higher in the trained limb than in the untrained limb (*P* = 0.05).
Shields et al., 2006 [54]	61%	**Participants:** 15 (5 chronic and 10 acute (C5-T10) SCI, **Age**:38±13.1,**Muscle(s)**: Soleus, **TSI**:8.79±1.85 years for Chronic SCI and within 6 weeks for Acute SCI	**Contraction:** Isometric. **Objective:** To quantify post-fatigue potentiation in the acutely and chronically paralyzed soleus and determine the effect of long-term electrical stimulation training on the potentiation characteristics of recently paralyzed muscle. **Study design:** While the participants remained in their wheelchairs, the ankle was stabilized in a system designed to measure the isometric plantar flexion torque of one leg. A stimulator was programmed to deliver a 10-pulse train (15 Hz; train duration 667 ms) every 2 s. The training protocol specified that 10,000 electrically stimulated contractions be completed each month (4 bouts of 125 contractions per day x 5 days x 4 week = 10,000 contractions). A bout of exercise consisted of 125 trains. Compliance was calculated as the percentage of the recommended number of contractions a participant completed in each month. **Stimulation**: PW: 667 ms, Frequency: 15 Hz, Amplitude: 0–200 mA	Participants in the training group attended laboratory-based stimulation sessions 1 to 4 times monthly identical with the home based training. Each participant completed 4 bout of exercise during each session with a 5 min rest between each bout. The protocol took 35 min to complete.	After 2 years of training, the bout 4 fatigue indices for the trained and untrained limbs were (82±1.08 and 62±4.3) % respectively (*P* = 0.05).Minimization of fatigue and post-fatigue potentiation during high-intensity FES activity for long duration (~35 min) was indicated.
Butler et al., 2004 [56]	52%	**Participants:** 8(C4-C6) SCI **Age**: 33±3**Muscle(s)**: Thenar, **TSI**:9.9±7.6	**Contraction:** Isometric. **Objective:** To compare the fatigability of the paralyzed human thenar muscles with and without a simultaneous voluntary contractions of the contralateral elbow flexor muscles. **Study design**: Each participant performed two experiments in random order, separated by at least one day. In both experiments, the fatigability of the thenar muscles was assessed. Experiment 1: involved an increase of thenar muscle perfusion pressure by increasing the systemic blood pressure when thenar muscle was fatigued. Experiment 2: involved no intervention during thenar muscle fatigue. **Stimulation:** PW: 667 ms, Frequency: 15 Hz, Amplitude: 0–200 mA	During pre-fatigue and fatigue protocol, a bag of weight (2.5-14Kg) were suspended (with the wrist while the elbow was flexed at ~ 90 deg.) while electrical stimuli were delivered and blood pressure was being monitored. The weight used was equivalent to 50% of the maximal one-repeat value.	Changes in mean arterial pressure (MAP) with exercise (median nerve stimulation with and without voluntary contractions) moderately correlated with changes in thenar muscle fatigue (*r*^*2*^ = 0.61; n = 7). For every 10% increase in MAP, fatigue was reduced by ~ 3%.
Gerrits et al., 2000 [44]	43%	**Participants:** 8 (C5-T8) SCI **Age**: 40.4±11**Muscle(s)**: Hamstring, Gluteal, and Quadriceps. **TSI**: 9.9±10	**Contraction:** Non-isometric. **Objective:** To assess if contractile speed and fatigability of paralyzed quadriceps muscles in persons with SCI can be altered by FES-LCE training. **Study design:** Contractile speed and fatigue characteristics of electrically stimulated isometric contractions were compared before and after 6 weeks of FES-LCE. When the pedalling rate dropped below 45 rpm, the resistance is reduced and when the pedalling rate dropped below 35 rpm, the stimulation was stopped. **Stimulation pattern:** PW: 450 μs, Frequency: 30 Hz, Amplitude: 140 mA to achieve target pedalling rate of 50rpm.	Participants trained for 30min, three times per week for 6 weeks.	Work output was significantly increased (4±5 kJ to 14±13 kJ; *P* = 0.019). At the end of 2min of stimulation, force declined to 32+10% of the pre-fatigue value and to 42+10% after training.
Gerrits et al., 2002 [55]	43%	**Participants**: 6 (C5-T12) SCI and 14 AB, **Age**: SCI: 41.8± 9.8AB: 27± 2**Muscle(s)**: Quadriceps **TSI**: 14.9± 8.1	**Contraction:** Isometric. **Objective:** To compare the effects of training with two patterns of FES (repetitive high-frequency stimulation (HFS) and more continuous low-frequency stimulation (LFS)) on the strength, contractile properties, and fatigability of paralyzed muscle. **Study design:** Both left and right quadriceps muscles of each participant were stimulated, one limb with the LFS protocol while the other was with the HFS protocol. LFS protocol, consisted of 35 min of repeated quadriceps activation with 10 Hz stimulation trains of 20 s duration, followed by a 50 s rest (duty cycle 28%). HFS protocol, consisted of 50 min of repeated stimulation, with 50 Hz trains of 2 s duration and followed by a 50 s rest (duty cycle 4%). Fatigability was assessed by activating the quadriceps muscle repetitively for 2 min at 30 Hz stimulation of 1 s duration every 2 s. **Stimulation pattern:** PW: 250 μs, Frequency: HFS- 50 Hz, LFS- 10 Hz, Amplitude: 130 ± 6 mA.	A daily bilateral isometric quadriceps contractions of legs for a period of 12 weeks.	Fatigue resistance increased significantly (*P*<0.05) from 2 weeks of LFS training (by 43%) but not (*P>*0.05) after HFS training even at 12th week of training.
Fornusek and Davis, 2004 [58]	48%	**Participants:** 9 (T4-T9) SCI **Age**: 37.8±10.4**Muscle(s)**: Hamstring, Gluteal, and Quadriceps. **TSI**: 3.12±0.91	**Contraction:** Non-isometric. **Objective:** To investigate the effect of pedal cadence upon torque production, PO and muscle fatigue rates during FES-evoked cycling. **Study design:** Trial 1: (n = 8) examined a low vs high pedal rate (20 and 50 rpm) upon isolated muscle fatigue over 5 min. Trial 2: (n = 9) investigated the effect of cadence (15 vs 50 rpm) upon performance during 35 min of FES-evoked cycling. Torque and PO were calculated by iFES-LCE while the quadriceps (300–30deg), hamstrings (60–160deg) and gluteal (6–73deg) muscle groups of both legs were stimulated to produce FES cycling. **Stimulation pattern: PW**: 250 μs, **Frequency**: 35 Hz, **Amplitude**: Trial 1–80 mA, Trial 2- (70–140 mA)	Each cadence was randomised and tested on a different day (Trial 1: 5 min, Trial 2: 35 min) with a maximum of 7 days between tests.	PT from the left quadriceps decayed significantly (*P*<0.05) faster at the higher pedal cadence. Cycling for over 35min showed that peak and average torques were significantly greater at the lower cadence. From 15min onwards, PO was significantly higher at 50rpm FES-cycling, compared with 15rpm (16.61± 1.14 kJ vs 13.21±1.16 kJ, *P*< 0.05).
Hartkopp et al., 2003 [57]	57%	**Participants:** 12 (C5-C6) SCI **Age**: 7(29–55) for high resistance (Hr) group 5(4–27) for low resistance (Lr) group, **Muscle(s)**: Wrist extensor, **TSI**: Hr: 5-38Lr: 4–27	**Contraction:** Isometric. **Objective:** To investigate the fatigue resistance capability of two different training protocols. **Study design:** The Hr group received 30 Hz stimulation with a duty circle of 5 s ‘on’ and 20 s ‘off’ against maximum load. The Lr group received 15 Hz stimulation with a duty circle increasing every third week. Total work output was similar in both protocols. The untrained arm was used as control. **Stimulation pattern:** PW: 250 μs, Frequency: 30 Hz, Amplitude: 140 mA to achieve target pedalling rate of 50 rpm.	The wrist extensor muscles were stimulated for 30 min/day, 5 days/week, for 12 weeks, using either a Hr or a Lr protocol.	PT at 15 Hz in the Hr group tended to improve by 19% (*P*<0.1), while Lr group did not improve. Fatigue resistance improved by 42% and 41% (*P*<0.05) in response to conditioning in the Hr group and Lr group, respectively.
Peckham et al.,1976 [53]	45%	**Participants:** 12 (C3-C7) SCI **Age**: 23.17±9.9**Muscle(s)**: Finger flexor, **TSI**: 1.98±5.05	**Contraction:** Isometric. **Objective:** To investigate whether the strength and endurance of electrically induced contractions in persons with SCI could be increased following FES exercise. **Study design:** The intramuscular nerves stimulation was modified to activate a relatively constant muscle fibre activation. The stimulation was surged on for a period of 2.5 s and off for the same duration. Exercise effectiveness was assessed by the contractile properties; the maximum tetanic force, the fatigue, and sometimes twitch characteristics. **Stimulation:** PW: 100 μs, Frequency: 10 Hz for 10 participants and 15 Hz for 2 participants. Amplitude: 20 mA maximum.	Stimulus evoked chronic exercise duration was up to 4 to 6 hours per day for 15±7 weeks in the hospital and at home.	A good trend *(r* = 0.808) for force development was reported. The correlation of exercise with fatigue changes was good *(r* = 0.827).The increase of force fatigue resistance was evident with maximum force after 25 weeks.
Sabatier et al., 2006 [60]	52%	**Participants:** 5(C5-C10) SCI **Age**: 36±5, **Muscle(s)**: quadriceps, **TSI**: > 5	**Contraction:** Isometric. **Objective:** To determine whether FES resistance training would (i) reduce muscle fatigability, (ii) reverse reduced arterial diameter, (iii) increase resting blood flow, and (iv) increase reactive and exercise hyperemia. **Study design:** Stimulus parameters were set to administer an equivalent of work to rest cycle of 5s on and 5s off. For the first 2 weeks, only the weight of the leg was used for the resistant traning while for another 16 weeks the load resistance increased by 0.9–1.8 kg/week. Muscle fatigue was calculated as a percent decline in torque production from the first 5 contractions to the last 5 contractions. **Stimulation pattern:** PW: 450 μs biphasic pulse and 50 μs phase delay, Frequency: 30 Hz, Amplitude: Current that was sufficient to elicited 30 Nm of torque	FES resistance training of knee extensions was performed twice a week for 18 weeks and measurements were made before training and after 8, 12, and 18 weeks of training.	A significant increase in weight lifted, as well as a 60% reduction in muscle fatigue (*P* = 0.001) was reported. The femoral arterial diameter range (*P* = 0.70), resting, reactive hyperemic, and exercise blood flow remained constant with training.
Gorgey et al., 2015 [59]	45%	**Participants:** 1T6 SCI, **Age:** 33, **Muscle(s):** Quadriceps, **TSI:** NM	**Contraction:** Non-isometric. **Objective:** To determine the effect of FES resistant training (RT) conducted once weekly on improving fatigue resistance regional and whole body composition. **Study design:** Quadriceps fatigue index was obtained from the number of repetitions (reps) achieved out of 30 reps. Total and regional body composition tests were conducted before the first session and one week after the last training session using whole-body dual-energy x-ray absorptiometry. **Stimulation pattern:** PW: 450 μs, Frequency: 30 Hz Amplitude: Range of 0- 200mA for each training set.	FES RT of the paralyzed quadriceps was conducted once weekly for 12 weeks using ankle weights.	The fatigue resistance of the right knee extensors increased from 56% at week 8 to 100% at week 11; while the left leg only achieved 80% in week 12.Once weekly of FES RT led to increased lean mass, strength, and fatigue resistance of trained knee extensors.

Abbreviations: SCI- Persons with spinal cord injury; PW- Pulse width; BMD: Bone mineral density; TSI: Time since injury; PT-Peak torque; TTi: Torque-time integral; iFES-LCE: Isokinetic functional electrical stimulation-Leg cycle ergometer, PO: Power output.

**Table 4:** Overview of included studies: Biofeedback-controlled stimulation.

Study	D&B Quality Rating	Population Age, TSI: years (Mean± SD)	Methods	Functional implications
Shields et al., 2006 [23]	48%	**Participants**: 8 (C6-T7) SCI, **Age**:40.25±15.65, **Muscle(s)**: Gastrocnemius and soleus **TSI**: ≤12	**Contraction**: Isometric. **Objective**: To determine the effectiveness of FDBCK controlled FES compared with the traditional stimulation modality. **Study Design**: An adaptive force feedback-controlled stimulation system (with consideration to safety, optimal overload and mechanism of fatigue) to monitor muscle torque during repetitive stimulation was developed. The fatigue protocol consisted of 125 contractions elicited by supramaximal stimulation of 10-pulses train (15 Hz) of the tibial nerve every 2 s for 667 ms. Participants volunteered for 3 or 4 stimulation sessions over a 6-month study period. **Feedback**: Drop in torque. **Stimulation pattern:** During 3 different stimulation protocols. High frequency protocol (H)Doublet protocol (D)Low frequency protocol (L)	From the 20th contraction to 125th, HDL and LDH FDBCK-FES outperformed the CF (*P* = 0.05). The mean torque and final torque of FDBCK-FES significantly outperformed that of CS FES (*P* = 0.05).
Dudley-Javoroski et al.,2011 [61]	48%	**Participants**: 13 (C5/C6-T12) SCI **Age**:34.4±10.7**Muscle(s)**: Quadriceps**TSI**:0.5–13.5	**Contraction:** Isometric. **Objective:** To compare the properties of paralyzed quadriceps force and femur compressive loads in an upright functional task during conventional constant-frequency stimulation and force feedback-modulated stimulation. **Study design**: participants performed 2 bouts of 60 isometric quadriceps contractions while supported in a standing frame. On separate days, participants received CONST at 20Hz or FDBCK triggered by a change in force. During FDBCK, a computer algorithm responded to each 10% reduction in force with a 20% increase in stimulation frequency. **Feedback:** Change in force. **Stimulation pattern:** Pulse: Sixty 100-Pulse trains (200 μs, 200 ms) Frequency: 20 Hz for CONST stimulation, and frequency modulated by FDBCK. Amplitude: 200 mA	Quadriceps peak force and fatigue index were higher for FDBCK than CONST (*P*<0.05).The decline in within-train force was greater during FDBCK bouts, but its mean force remained above that of CONST (*P*<0.05).As fatigue developed during repetitive stimulation, FDBCK was superior to CONST for maintenance of femur compressive loads.

Abbreviations: SCI- Persons with spinal cord injury; CONST Constant frequency stimulation; FDBCK: Feedback-controlled stimulation; TSI: Time since injury; TTi-Torque-time integral; CF-Open loop constant frequency; HDL- (H), (D) and (L) trains given in succession; LDH-(L), (D) and (H) trains given in succession.

### Methodological quality

The quality of the included studies was moderate *i*.*e*. meeting up to an average of 50% of the criteria (range: 38–70%, Table 5) on the D&B scale. None of the studies reported the following criteria; adverse events that may be consequence of the intervention (criterion 8), concealed allocation and blinded participants, therapists and assessors (criterion 14 and 15), adjustment to the effects of confounders in the data analysis (criterion 25). Among controlled studies (studies with treatment and controlled groups [32]) only that of Downey et al., 2014 [33] partially described the distribution of principal confounders which may account for differences between the treatment and control groups. The criteria commonly unfulfilled were; the reporting of exact *P* values (criterion 10) and randomisation of study participants to the intervention groups (criterion 23). However, all studies specified the eligibility criteria, and measured key outcomes in up to 100% of the initial participants except Godfrey et al., 2002 [34] and Scott et al., 2005 [14] which derived the outcomes from 50% and 67% of the recruited participants, respectively.

**Table 5:** Methodological quality of included studies based on Downs and Black** checklist (n = 35).

Study	1	2	3	4	5	6	7	8	9	10	11	12	13	14	15	16	17	18	19	20	21	22	23	24	25	26	27	Quality Score	Percentage (%)
Karu et al., (1995)	1	1	1	1	0	1	1	0	N/A	1	0*	0*	1	0	0	1	N/A	1	0*	1	0*	N/A	0	0	0	0*	N/A	11/24	46
Graupe et al, (2000)	1	1	0	1	N/A	1	1	0	N/A	0	0*	0*	1	0	0	1	N/A	0*	0*	1	0*	N/A	N/A	0	0	0*	N/A	8/21	38
Griffin et al., (2002)	1	1	1	1	0	1	1	0	N/A	1	0*	0*	1	0	0	1	N/A	1	0*	1	0*	N/A	0	0	0	0*	N/A	11/24	46
Godfrey et al., (2002)	1	1	1	1	N/A	1	1	0	N/A	1	0*	0*	1	0	0	1	N/A	1	0*	1	0*	N/A	0	0	0	0*	N/A	11/22	50
Eser et al., (2003)	1	1	1	1	N/A	1	1	0	N/A	1	0*	0*	1	0	0	1	N/A	1	0*	1	0*	N/A	1	0	0	0*	N/A	12/22	55
Thomas et al., (2003)	1	1	1	1	0	1	1	0	N/A	1	0*	0*	1	0	0	1	N/A	1	0*	1	0*	N/A	0	0	0	0*	N/A	11/24	46
Kebaetse et al., (2005)	1	1	1	1	N/A	1	1	0	N/A	0	0*	0*	1	0	0	1	N/A	1	0*	1	0*	N/A	1	0	0	0*	N/A	11/22	50
Decker et al., (2010)	1	1	1	1	N/A	1	1	0	N/A	1	0*	0*	1	0	0	1	N/A	1	0*	1	0*	N/A	1	0	0	0*	N/A	12/22	55
del-Ama et al., (2012)	1	1	1	1	0	1	1	0	N/A	1	0*	0*	1	0	0	1	N/A	1	0*	1	0*	N/A	1	0	0	0*	N/A	12/24	50
Chang and Shields, (2011)	1	1	1	1	0	1	1	0	N/A	0	0*	0*	1	0	0	1	N/A	1	0*	1	0*	N/A	0	0	0	0*	N/A	10/24	42
Graham et al., (2006)	1	1	1	1	0	1	1	0	N/A	1	0*	0*	1	0	0	1	N/A	1	0*	1	0*	N/A	1	0	0	0*	N/A	12/24	50
Thrasher et al., (2005)	1	1	1	1	N/A	1	1	0	N/A	1	0*	0*	1	0	0	1	N/A	1	0*	1	0*	N/A	1	0	0	0*	N/A	12/22	55
Bickel et al., (2004)	1	1	1	1	0	1	1	0	N/A	0	0*	0*	1	0	0	1	N/A	1	0*	1	0*	N/A	0	0	0	0*	N/A	10/24	42
Scott et al., (2007)	1	1	1	1	N/A	1	1	0	N/A	0	0*	0*	1	0	0	1	N/A	1	0*	1	0*	N/A	1	0	0	0*	N/A	11/22	50
Scott et al., (2005)	1	1	1	1	N/A	1	1	0	N/A	0	0*	0*	1	0	0	1	N/A	1	0*	1	0*	N/A	1	0	0	0*	N/A	11/22	50
Deley et al., (2015)	1	1	1	1	N/A	1	1	0	N/A	1	0*	0*	1	0	0	1	N/A	1	0*	1	0*	N/A	1	0	0	0*	N/A	12/22	55
Gorgey et al., (2014)	1	1	1	1	N/A	1	1	0	1	1	0*	0*	1	0	0	1	N/A	1	1	1	1	N/A	1	0	0	1	N/A	16/23	70
Chou et al., (2008)	1	1	1	1	N/A	1	1	0	N/A	0	0*	0*	1	0	0	1	N/A	1	0*	1	0*	N/A	1	0	0	0*	N/A	11/22	50
Popovic and Malesevic, (2009)	1	1	1	1	N/A	1	1	0	N/A	0	0*	0*	1	0	0	1	N/A	0*	0*	1	0*	N/A	0	0	0	0*	N/A	9/22	41
Malešević et al., (2010)	1	1	1	1	N/A	1	1	0	N/A	0	0*	0*	1	0	0	1	N/A	1	0*	1	0*	N/A	0	0	0	0*	N/A	10/22	45
Nguyen et al., (2011)	1	1	1	1	N/A	1	1	0	N/A	1	0*	0*	1	0	0	1	N/A	1	0*	1	0*	N/A	1	0	0	0*	N/A	12/22	55
Sayenko et al., (2014)	1	1	1	1	N/A	1	1	0	N/A	1	0*	0*	1	0	0	1	N/A	1	0*	1	0*	N/A	0	0	0	0*	N/A	11/22	50
Downey et al., (2014)	1	1	1	1	1	1	1	0	N/A	1	0*	0*	1	0	0	1	N/A	1	0*	1	0*	N/A	1	0	0	0*	N/A	13/24	54
Shields and Dudley-Javoroski (2006)	1	1	1	1	N/A	1	1	0	0	0	0*	0*	1	0	0	1	N/A	1	1	1	0*	N/A	0	0	0	0*	N/A	11/23	48
Shields et al., (2006)	1	1	1	1	N/A	1	1	0	1	1	0*	0*	1	0	0	1	N/A	1	1	1	0*	N/A	0	0	0	1	N/A	14/23	61
Butler et al., (2004)	1	1	1	1	N/A	1	1	0	0	1	0*	0*	1	0	0	1	N/A	1	0	1	0*	N/A	1	0	0	0	N/A	12/23	52
Gerrits et al., (2000)	1	1	1	1	N/A	1	1	0	0	1	0*	0*	0	0	0	1	N/A	1	0*	1	0*	N/A	0	0	0	0*	N/A	10/23	43
Gerrits et al., (2002)	1	1	1	1	N/A	1	1	0	0	0	0*	0*	0	0	0	1	N/A	1	0*	1	0*	N/A	1	0	0	0*	N/A	10/23	43
Fornusek and Davis, (2004)	1	1	1	1	N/A	1	1	0	0	0	0*	0*	1	0	0	1	N/A	1	0*	1	0*	N/A	1	0	0	0	N/A	11/23	48
Hartkopp et al., (2003)	1	1	1	1	N/A	1	1	0	1	0	0*	0*	1	0	0	1	N/A	1	0*	1	0*	N/A	1	0	0	1	N/A	13/23	57
Peckham et al., (1976)	1	1	1	1	N/A	1	1	0	0	0	0*	0*	1	0	0	1	N/A	1	0*	1	0*	N/A	N/A	0	0	0	N/A	10/22	45
Sabatier et al., (2006)	1	1	1	1	N/A	1	1	0	0	1	0*	0*	1	0	0	1	N/A	1	1	1	0*	N/A	0	0	0	0*	N/A	12/23	52
Gorgey et al., (2015)	1	1	1	1	N/A	1	1	0	0	0	0*	0*	0	0	0	1	N/A	1	1	1	0*	N/A	N/A	0	0	0*	N/A	10/22	45
Shields et al., (2006)	1	1	1	1	N/A	1	1	0	0	0	0*	0*	1	0	0	1	N/A	1	0	1	0*	N/A	1	0	0	0	N/A	11/23	48
Dudley-Javoroski et al., (2011)	1	1	1	1	N/A	1	1	0	0	0	0*	0*	1	0	0	1	N/A	1	0	1	0*	N/A	1	0	0	0	N/A	11/23	48

Abbreviation: D&B criteria met = 1, D&B criteria not met = 0, Unable to determine = *0, i.e. scored 0, Criteria is not applicable to the study = N/A, Unable to determine = UTD.

The table presents the grading of included studies based on the D&B criteria 1 to 27.

Downs and Black**- Criteria are as summarised below

1-Hypothesis/aim stated, 2- Outcome described in introduction/ method, 3- Participants’ characteristics described, 4- Intervention described, 5- Distribution of principal confounders described, 6- Findings described, 7- Data distribution, 8- Description of adverse events, 9- Characteristics of participants’ lost to follow-up described, 10- Exact *p*-value reported, 11- Participants’ sources described, 12- Participants’ selection described, 13- Appropriateness of the experimental facility, 14- Blinding of the participants to the intervention, 15- Blinding of the examiner, 16- “Data dredging”, 17- Analysis adjustment to different length of follow-up, 18- Appropriate statistics used to measure outcomes, 19- Adherence to the intervention, 20- Accuracy of outcome measures, 21- Source of participant for comparison groups, 22- Time period of participants’ recruitment, 23- Participants’ randomisation, 24- Randomised intervention assignment, 25- Adjustment for confounders during analysis, 26- Consideration to loss to follow-up, 27- Statistical power of the outcome of the study.

Criterion 5 has a maximum of 2 points while others have a maximum of 1 point each. Points were awarded only when the criteria were clearly described.

### Study characteristics

Out of the 35 included studies, thirty-one were peer-reviewed journal articles, three were case studies or pilot investigations, and one conference paper. There were no RCTs in the scope of interest, and most studies lack control conditions. Subject heterogeneity and diverse methodology partially limited a quantitative or meta-analytical comparison between studies, and amongst strategies. Most of the studies were published from 2005 to 2014 highlighting an increased interest recently in this field.

### Modification of stimulation patterns

Functional electrical stimulation to evoke muscle contractions is typically effected through the modification of key stimulus parameters (*i*.*e*., pulse width, frequency, amplitude) [35], the effectiveness of which could be graded by their ability to offset rapid muscle fatigue. The effectiveness of optimal activation strategies comprising stimulation patterns and modulation of different stimulation trains for reducing muscle fatigue in persons with SCI (Table 1) were investigated in eighteen studies.

Karu and colleagues [35], originally demonstrated that a strategy of *N*-let pulse trains (*i*.*e*., a set of closely spaced doublet stimulation pulses) showed a significantly longer torque time integral (TTi) when compared to the traditional single-pulsed stimulation. This stimulation pattern enabled the optimization of force production per stimulation pulse. However, the small sample size of this study and a marked between-subject variability led to the heterogeneity of responses amongst individuals. Two studies, one by Griffin et al., [36] with matched sample sizes and the other by Chang and Shields [37] with wider sample size demonstrated that the doublets pulse pattern was more effective for producing and sustaining force in paralyzed thenar and soleus muscles, respectively. Another four studies considered the effects of modulation of different pulse trains on the force augmentation. Switching frequency trains (*i*.*e*., from a constant frequency trains (CFT, where six 200 s square wave pulses separated by 70 ms were used) to a double frequency train (DFT, where a train of pairs of closely spaced pulses (doublets, 5ms) separated by longer intervals were used)) demonstrated improvements in fatigue resistance [14], whereas administration of CFT and DFT separately were less optimal [21]. In addition, during paralyzed quadriceps’ strength training, Deley et al., [20] verified that variable frequency train (VFT, where a train identical to the CFT were used, except that the first 2 pulses were separated by 5 ms) stimulation pattern and VFT followed by CFT generated less fatiguing contractions. Taken together, these findings suggest that the frequency modification of different stimulation trains offsets rapid muscle fatigue.

In contrast, Thomas et al., [38] evaluated the fatigue-reducing ability of variable versus constant frequency pulse trains in paralyzed thenar muscles and observed that both patterns did not significantly reduce muscle fatigue of functional relevance unless complemented with other fatigue reducing strategies. Bickel and co-workers [39] reported that the use of VFT may not be efficacious to enhance torque during fatiguing contractions. The authors identified that the fast contraction rise time that was evident by inability of the inter-pulse interval to augment the peak torque might be responsible. They observed that the mechanism of fatigue and its management may be dissimilar in able-bodied and SCI populations. However, pre-conditioning of the SCI muscles was recommended for future applications of pulse train modulation [39]. Recently, Gorgey et al. [40], examined the effect of modulating the stimulation pulse width (while the frequency value remained constant (CFT)) on the fatigability of paralyzed muscle following cycling exercise. The investigators reported that modulating pulse width could not influence the muscle fatigability significantly. Based on the available evidence [21, 40], there is no significant effect of only CFT on the reduction of rapid fatigue in paralyzed muscle. Thus, the traditional method of keeping the stimulation frequency constant might not be an appropriate strategy to reduce muscle fatigability.

During repetitive electrical stimulation, progressive increases of stimulation frequency and intensity (PIFI) has been shown to generate extended contractions [41]. Kebaetse and colleagues [12] in their study, started with a low frequency and switched to a higher frequency stimulation. They were able to produce a repetitive, non-isometric contractions in persons with SCI by minimizing the rate of fatigue during a low-frequency stimulation phase. Eser et al., [42] and del-Ama and co-workers [43] observed that frequency modulation could promote extended contractions during cycling and isometric exercise and should be further investigated as a strategy to regulate power output and subvert muscle fatigue. In addition to using isometric contraction to assess fatigue characteristics (*i*.*e*., muscle force and work output) following FES cycling [44], isometric contraction exercise promotes prolonged contraction necessary to augment power output and extend cycling time.

A case study by Graupe and co-workers [45], demonstrated an improvement of FES standing and walking by random stimulation of inter-pulse interval (as against the constant frequency stimulation) in a range of ±12% between FES pulses. The random stimulation of inter-pulse interval was meant to excite action potential in the peripheral nerves resulting in nerve membrane depolarization to produce extended muscle contractions [45]. Although the study could not be generalised to a wider population due to a single case study design, it served as a platform for further systematic investigations to extend the standing and walking duration in a larger test group.

In contrast, Graham et al., [46] and Thrasher et al., [8] independently evaluated four modes of stimulus parameter modulation and observed that the random modulation of stimulus parameters may not be a viable strategy for fatigue resistance during FES-induced contractions. Although the authors suggested that the 10 min rest between trials might be insufficient for full restoration of muscle strength, subjects’ heterogeneity might also have been responsible for their observations. Based on these evidence, the modulation of FES parameters including pulse width, frequency, and amplitude has therefore, been identified as an essential strategy used to mimic the natural MU recruitment patterns and promotes reduced fatigability [41].

A study by Godfrey and colleagues, [34] advocated for submaximal stimulation to delay muscle fatigue. However, the study was marked with inconsistences in the level of muscle fibre recruited across the subject to the extent that the investigators had to adopt the data of the half of the subjects for the analysis (*i*.*e*., those that were near to each other). This limitation may be responsible for the lack of popularity of this strategy.

A study by Decker and colleagues [47] investigated the validity of altering stimulation strategy over the traditional ‘co-activation’ strategy in delaying muscle fatigue during cycling. Authors identified that the alternation of stimulation among muscles offers an enhanced benefit to FES therapy because the strategy distributes activation among a set of synergistic muscles and delay fatigue by allowing individual muscle to rest during the deactivation periods [47].

Apart from a preliminary study by Graupe and colleagues [45] which reported a delay of rapid fatigue during standing and walking, that by Decker and co-workers [47], Eser et al., [42], and Gorgey et al., [40] which investigated the possibility of rapid fatigue offset during FES cycling, other investigations have only considered either improvement in the muscle force/torque or endurance during FES-evoked isometric contractions. Interestingly, there is evidence of similar cardiorespiratory responses following both isometric and cycling contractions [48]. Thus, other than promoting reduced fatigability, intermittent isometric contraction might be an equally viable alternative as cycling for improving the whole body metabolic rate during FES exercise in persons with SCI [48].

Based on the available evidence, the effectiveness of modification of stimulation pattern to prevent rapid muscle fatigue is typically assessed through comparison of the fatigue index (*i*.*e*., final torque normalized to maximum torque), fatigue time (*i*.*e*., time for torque to drop by 3 dB) [46], and torque-time integral (*i*.*e*., over the entire trial). The administration of the optimal parameters of stimulation appears to be essential for the sustenance of FES-induced functional activity. In summary, the potential for minimizing rapid muscle fatigue during FES-evoked functional activities through the modulation of neuromuscular stimulus parameters is quite evident, and this remains an important research domain for optimization of FES therapy [40, 43].

### Optimization of electrode positioning

Five studies investigated the importance of electrode position to reduce the fatigue characteristics of FES-induced muscle contractions in SCI population (Table 2). Popovic and Malesevic [49] demonstrated an increased mean fatigue interval by 153% in a multi-electrode array as compared with a single cathode electrode. Malesevic and colleagues [50] confirmed that a low pulse frequency (four smaller cathodes at 16 Hz each) was better suited for a prolonged stimulation with a stronger, and fused tetanic contractions when compared to a high pulse frequency (single cathode at 30 Hz), although at the expense of force production [50]. Equally, the spatially distributed sequential stimulation configuration has demonstrated a greater fatigue reducing ability compared to a single active electrode stimulation configuration in the upper limbs [51] and lower limbs [52]. This is because different sets of muscle fibres are activated alternatively by different electrodes, enabling activation of sub-compartment muscles [52]. Similarly, Downey and co-workers [33] demonstrated a significant reduction of FES-induced fatigue with an asynchronous stimulation protocol. Although stimulation electrode placement over the peripheral nerve trunk and motor points are frequently cited locations, a general consideration for electrode placement appears to be a function of the intended purposeful activity. Moreover, available evidence have identified the possibility of significant inter-individual variability in the location of motor points and that an overlap of the electrode must be avoided [51].

In general, the proper placement of stimulation electrodes over motor points of the target muscle is necessary for optimal muscle fibre recruitment. Additionally, the use of multi-electrode cathode has been recommended over a single cathode electrode. Clear understanding of the anatomical landmarks where sufficient motor units maybe recruited has been identified as an important precursor for selective recruitment of fatigue-resistant motor units. Thus, the application of multi-electrode stimulation arrays has not only been applied to selectively recruit the motor unit for efficient motor control, but has equally been validated to be more effective to offset rapid muscle fatigue.

### Optimization of exercise training

While some previous studies have sought to improve fatigue-resistance via technical or technological approaches to neuromuscular stimulation, such as modulating neuromuscular pulse trains or deploying multi-electrode arrays, less research has addressed whether better fatigue-resistance might be achieved by improved muscle training paradigms for patients. Rehabilitation of persons with SCI involves continuous training and exercise in order to achieve the desired outcomes via task specific training [22]. Ten studies assessed the effect of exercise on the fatigue characteristics during FES-induced contractions in SCI population. A study by Peckham and colleagues [53] investigated whether FES-induced contractions of finger flexors of persons with SCI could elicit usable force and endurance necessary for functional hand grasp. They reported an increase of force development and fatigue resistance following FES training oriented on functional activity. Although there were significant variations in the participants’ responses in terms of force production and fatigability, the study generally demonstrated the possibility of modification of contractile properties of paralyzed human skeletal muscles through FES exercise [53]. Two independent studies by Shields and Dudley-Javoroski [22] and Shields and co-workers [54] showed an increase in endurance time and fatigue resistance following two years of isometric contractions of the ankle plantar flexors [22] and prevention of post fatigue potentiation in soleus muscles [54] respectively. Authors further suggested that electrical stimulation may be used to predict contractile characteristics in paralyzed muscles. Another study by Gerrits et al., 2002 [55] compared the effects of leg isomeric training using two patterns of FES (*i*.*e*., repetitive high-frequency stimulation (HFS) and more continuous low-frequency stimulation (LFS)) on the strength, contractile properties, and fatigability of paralyzed quadriceps muscle. Authors showed that fatigue resistance increased significantly (*P*<0.05) from 2 weeks of LFS training (by 43%) but not (*P*>0.05) with HFS training even after 12 weeks of training.

Butler et al., [56] evaluated the effect of blood pressure control during exercise and found that improved blood pressure in thenar muscles was moderately correlated to fatigue resistance. However, investigators mentioned certain difficulties and safety associated with raising the mean arterial pressure in persons with SCI as the major concern with this strategy. Similarly, Hartkopp and colleagues, [57] compared the fatigability of trained wrist extensors to high frequency stimulation (30 Hz) and low frequency stimulation (15 Hz). Although the two protocol significantly offset the rapid fatigue, only high frequency protocol considerably augmented muscle force and aerobic metabolism after training [57]. Thus, strategies that promote improved aerobic metabolism could lengthen contraction time during FES exercise.

Owing to the difficulty of translating the neurological changes into functional outcomes, rehabilitation intervention in persons with SCI has been focused extensively on functional training. Improved adaptable response and fatigue resistance ability of paralyzed muscles to functional training have been demonstrated in FES induced cycling. Gerrits et al., [44] evaluated the contractile speed and fatigability of paralyzed quadriceps muscle to the FES leg cycle ergometry (FES-LCE) exercise. Increased fatigue resistance was reported as indicated by the higher force maintained. However, authors reported large variability in fatigue resistance between participants that was also negatively correlated with the time since injury (TSI) of the participants [44]. This suggests the need for a consideration of the injury levels and TSI while selecting FES patterns for therapy. Similarly, Fornusek and Davis [58] investigated the effect of pedalling cadence upon torque production and muscle fatigue during FES cycling. Although the investigators identified significantly better delay of leg muscle fatigue during low cadence pedalling, high cadence produced better power output [58]. The authors expressed this as a trade-off in a muscle’s force-velocity profile—an electrically stimulated muscle group may produce a higher power output at a higher cadence but with rapid fatigue, or lower power output at a slower cadence with reduced fatigability. The need to further investigate the relative contribution of stimulation period and leg angular velocity to different fatigue rates was also suggested.

Two independent studies [59, 60] reported significant effects of FES resistance training (RT) on paralyzed muscle fatigability. Specifically, Gorgey et al. [59], in a case study, showed the possibility of once a week FES RT to offset rapid quadriceps muscle fatigue. Although being a single case report, the result could not be generalized, but a proof of concept on a simple method to sustain regional muscle adaptations to training could be inferred. Similarly, Sabatier and colleagues [60] reported 60% reduction in quadriceps muscle fatigue following 12 weeks of FES RT in five persons with SCI. These authors proposed that FES resistance training could increase muscle size and promote reduced denervated muscle fatigability.

In the interim, FES exercise therapy in persons with SCI is of considerable clinical value, especially as it increases contraction time and fatigue resistance due to the high percentage of fatigue-resistance fibres associated with the loading effect following exercise [22]. FES exercise has also been shown to promote plasticity, hypertrophy and endurance due to the considerable neural adaptation [22, 27]. Although it has sometimes been claimed that FES exercise training transcends just fatigue resistance but also may promote neuroplasticity, there are few studies that have substantiated this in the SCI population (Table 3). There may be differences between fatigue responses in chronic and acute SCIs due to the variations in the duration of inactivity associated with the changes in muscle metabolism, blood flow, and fibre composition [53]. The marked differences between the exercise responses in chronic and acute SCI seemed to have been overlooked in all the studies under this category. In summary, timely and appropriate FES-evoked exercise training in persons with SCI has been frequently cited to significantly impact fatigue resistance [22]. Future research must focus on which components of the FES-exercise prescription (intensity, duration or weekly frequency) are most ‘dose-potent’ for functional and health outcomes to patients.

### Biofeedback-controlled stimulation

The effectiveness of recent application of biopotential-controlled FES exercise in reducing rapid fatigue during FES training was assessed by two studies [23, 61]. Shields and colleagues [23] compared the effectiveness of online modulation (closed loop) and traditional modality (open loop) and found that online modulation of stimulation parameters (*i*.*e*., through feedback controller to regulate muscle torque) significantly enhanced the generated gastrocnemius and soleus muscles’ mean and peak torques even when the pattern of stimulation was randomised [23]. Similarly, Dudley-Javoroski and co-workers, [61] reported significant improvement in the quadriceps peak force and fatigue index with the application of biofeedback controlled FES as compared to the traditional open loop FES. In summary, incorporation of biofeedback to control FES has been shown to impact fatigue resistance positively and enhanced physiologic performance of paralyzed muscles [61]. Integration of biofeedback into FES technique is promising and sensor technology for measuring biopotentials may revolutionize the scope of the FES technology if adequately substantiated in clinical populations.

## Discussion

This systematic review has established that previous studies demonstrated only partial success for ameliorating rapid muscle fatigue during FES muscle contractions in persons with SCI. Muscle force and its derivatives were identified as the key indices of fatigue. The available evidence revealed a number of important strategies often cited to delay the onset of muscle fatigue including the optimization of electrode positioning, modification of patterns of stimulation and its parameters, optimization of the mode and frequency of exercise training, and biofeedback-controlled FES exercise. These modalities generally resulted in selective recruitment of fatigue resistant motor units [34, 62]. The literature also supported the use of increase in endurance time during repetitive contractions as indication of muscle fatigue resistance in persons with SCI. However, limited evidence was available concerning the direct investigation of the delay of muscle fatigue during FES-assisted functional activities such as cycling [40, 42, 44, 47, 58], or standing and walking [45] in persons with SCI. Apparently, overcoming the limitations imposed by this gap of knowledge may be an essential element in promoting FES assisted activities of daily living.

The long-time goal of FES technology has been to restore functional tasks and form an integral part of SCI management. A major step in this direction is to identify fatigue resistance strategies in order to achieve an effective therapy. Modification of stimulation parameters has been mostly explored among the strategies identified. Out of eighteen studies that sought to titrate neuromuscular stimulus parameters, Graupe and colleagues [45] was a preliminary investigation, while Karu et al., [35] reported a significant within-subject variation. del-Ama and co-workers [43] clarified the limitation of their experimental findings only to the quadriceps isometric contractions during stance phase of walking. Inability of the SCI patients to develop adequate force required for swing phase was identified as a hindrance to isokinetic test in their approach [43]. Caution should be exercised in the future application of these methods due to their low methodological quality. Nevertheless, all the studies except two [8, 46] reported modification of stimulus parameters as a significant fatigue-reducing strategy. The two studies with dissimilarity identified that their results might be due to the insufficient rest period between trials [8, 46]. This suggests significant implication of adequate resting period in muscle fatigue assessments and reduction studies as well as the validity of modification of stimulation parameters, particularly stimulation frequency for rapid muscle fatigue reduction in FES-based rehabilitation interventions.

Peckham et al., [53] originally proposed exercise regimen induced by chronic electrical stimulation for fatigue resistance based on the inability of FES to develop sufficient force to sustain muscle contractions in paralyzed muscles. However, exercise interventions can only be effective if the electrical stimulation is robust and applied in such a way that it is substantially resistance to rapid muscle fatigue. In addition to other physiological benefits of FES exercise during rehabilitation, evidence suggests that exercise offsets rapid muscle fatigue and promote a better rehabilitation outcome in the clinical populations [11]. However, a large number of relevant studies [22, 44, 53, 56, 58] under this category failed to report any of the characteristics of participants lost to follow-up. Thus, there is a need to intensify efforts on home-based FES systems and the prescription of training ‘dose’ to facilitate self-care post SCI [22], since there may be significant time constraints for patients to attend clinics and gymnasiums.

Although the physical deconditioning secondary to SCI can be favourably altered by exercise interventions, some authors have argued that improvement of physical functionality does not entirely rely on exercise since the restoration of functional capacity depend on many other factors [63]. Among the factors that alter skeletal muscle morphology and function are severity of loss of voluntary motor control [63] and the level of injury [64]. It is well acknowledged that the ambulation and independence prognosis in the activities of daily living is primarily predicted by the level of injury and the completeness of the SCI [65]. Thus, the consequent variation in the motor and sensory functions in different SCI populations alters muscle contractile properties and significantly affect force-generating and fatigue-resistance capacity [65]. Therefore, investigation of the response of available strategies (with consideration to the effect of level and completeness of injury), on muscle fatigability needed to be understood in order to adequately position the clinical implication of the strategies.

Accordingly, an extensive muscle training to promote conditioning should be complemented with other regimens with consideration to specific level and completeness of injury in order to gain useful insight into key physiological functions necessary to improve muscle fatigue resistance. In all, reduction of rapid muscle fatigue during FES therapy in persons with SCI, being a complex task, has been managed using multiple, often concurrent, strategies. However, these are still mostly confined to the research laboratories based on the settings of most studies we reviewed. An integrated approach towards fatigue reduction may allow a successful deployment of the strategies for therapeutic trials in clinical settings and outdoor where the full potential of FES therapy can be derived. Currently, comparisons between the effectiveness of each of these strategies are difficult because of the lack of standardisation of the protocols, training procedures and stimulation parameters administered in different studies even within the same category of strategy. Accordingly, the immense potentials in the generation of fatigue resistant contractions bespeak the importance of randomised controlled trials (RCTs) to document the efficacy of available strategies. While further research is apparently required, preliminary data on the available strategies are promising. Although there is significant impact of each strategy which could serve as basis for further investigations, a combination of the strategies appears to be more effective.

Furthermore, it is axiomatic that the earlier the FES therapy commences following the injury the better will be the outcome, and the associated inactivity-atrophic changes in paralyzed muscles are reversible even after 20 years [10]. Apart from the study by Bickel et al., [39] and that of Chang and Shields [37], subjects with complete and incomplete lesions [49, 50], as well as those with acute and chronic injuries [54] were grouped together during fatigue reduction studies. This might prevent the understanding of specific influence of fatigue reduction strategies on the time since injury—since skeletal muscles have different fatigue resistance capacity during post-SCI stages [51]. For example, although denervated muscles generally show disuse atrophy, in chronic SCI such atrophy is concomitant with an increase in the interstitial endomysial connective tissues and perifascicular fatty infiltration [66], attributable to muscle inactivity [67]. These profound morphological changes impact motor unit orientation and muscle fatigability significantly. Moreover, unlike during chronic SCI, an acutely denervated skeletal muscle might be characterized by an unusual muscle fibre composition (as indicated by the relative proportion of slow and fast myosin heavy chain isoform expression) [68], particularly during alteration of fibre type morphology and histochemistry after SCI [40, 68]. These findings suggest different force-fatigue temporal responses (between acutely-denervated versus chronically-denervated muscles) to different fatigue reduction strategies. Therefore, integration of all the strategies in optimizing muscle performance should be further assessed in clinical populations with due consideration to “time since injury” in order to fully appreciate the functional benefits associated with the strategies.

Evidence indicates that most fatigue reduction investigations were conducted during isometric contractions of skeletal muscles. However, validation of those strategies during non-isometric conditions which are observed within functional tasks will be more clinically relevant. Equally, few muscle groups such as quadriceps and thenar have been evaluated, the test on the other functionally relevant muscle groups will determine how broadly the current strategies can be generalized. Therefore, further research trials are required before a definitive conclusion can be drawn on the effectiveness of FES fatigue reducing strategies in clinical population during functional activities. In all, the relationship among the strategies seems to be complementary [51], since there is not one without some limitations [8]. The lack of sufficient data to advance an external validity on a generalised mode of stimulation is conceivable as each FES candidate often present a unique clinical picture and different responses to a specific stimulation pattern. Lack of obvious methods of data extraction, different experimental settings and subject heterogeneity might have informed the considerable variation of the choice of fatigue measurement used in the studies reviewed.

### Future directions

Despite an extensive evidence-based knowledge establishing the decreased fatigue resistance following SCI, little research has been performed to subvert this trend. Therefore, the prevalent incidence of SCI could not be matched with the studies in this field. Available efforts to automatically minimize this phenomenon remained limited, creating a wide knowledge gaps for the development of an ideal FES device. There may be device dependent limitations in FES therapy due to the manifestation of innate nature of the FES device and its effect on muscle physiology (*i*.*e*., unnatural recruitment patterns). The emerging trend is tending toward a systematic approach to the administration of the optimal stimulation parameters [37] and identification of exercise dose potency necessary to augment the outcomes [22]. These strategies may require sophisticated FES devices in terms of number of channels, signals controls and portability.

Another concern in muscle fatigue management beyond FES device is the interfacial relationship between the device and the muscle of interest. Important advancements in the area of rapid muscle fatigue reduction includes the stimulation at the motor point innervating the target muscles [69]. At the moment, the studies under this category [33, 49–52] have limited functional applications as they were conducted during isometric contractions. The electrophysiological procedure recommended by Gobbo and colleagues [69] on able-bodied should be further validated in SCI population during functional tasks. This is because the result of the investigations on able-bodied volunteers may not be simply or directly applied to SCI cohorts [21].

As identified from the literature, studies on the application of FES are marked with heterogeneity of stimulation patterns that may be responsible for the wide variation of outcomes. This problem may be partly ameliorated by an automated biofeedback-controlled FES which could be used to control the stimulation parameters to suit the fatigue state of different muscles. Development and validation of sensors to modulate and automate the stimulation pattern in FES muscle contractions in order to mimic the physiological coordination of muscular activities remain a wide knowledge gap. Indirect measures of neural activities including electromyogram (EMG) [70], electroencephalogram (EEG), electroneuragram (ENG) [71] and mechanomyogram (MMG) [47, 72] (*i*.*e*., indices of neural information to decode functional intentions) have been validated as physiological sensing modalities. These sensors are promising in the design of biofeedback systems to achieve a ‘closed looped’ FES control in clinical populations [73]. They may be deployed to serve as proxy of muscle contractions (*i*.*e*., generated muscle force during fresh and fatiguing contractions), and the knee-joint dynamics [74] for the development of biopotential feedback controllers during FES-induced contractions.

There are indications of improved FES exercise outcome in SCI populations if combined with dietary supplementations. Nutrition such as whey protein and carbohydrate supplement [75], and other supplementations including creatine [76] have been proposed to augment functional responses (*i*.*e*., by boosting the lactate threshold during high intensity exercise) and promote endurance, time to fatigue and calorie expenditure during FES exercise. However, the available studies are marked with limitations including investigation on small case series with no control groups and were conducted without consideration to other circulatory dysfunction following SCI (*i*.*e*., which may have contributed to the fatigue patterns observed) [75]. Apart from these limitations, there is inadequate awareness on the precise mechanism of actions and side effects of these dietary intakes [77]. Nevertheless, there is an encouraging trend that these supplementations may enhance exercise capacity in SCI populations. Further investigations to quantify its short and long-time effects in promoting endurance, and reducing time to fatigue will be enlightening.

### Study limitations

The results of this review are limited to studies written in English language and included in the databases used to identify the articles. Other studies may exist outside these domains which we were unable to retrieve. Selection bias is another potential limitation of systematic reviews. However, we attempted to minimize the bias by searching for both randomized and non-randomized studies and stating clearly the quality of each study. Incidentally, there was no randomized controlled trial in the scope of interest. This was assessed independently by two reviewers. There may be other limitations beyond our control such as publication bias. Frequently, only studies with positive results are submitted to peer reviewed journals for publication. This situation may bias results toward positive treatment effects. We endeavour to widen the search scope from the earliest time to the most recent, June 2015 to limit such effect. Nevertheless, an interesting trend in rapid fatigue management could still be objectively inferred.

## Conclusion

The strategies for the rapid muscle fatigue reduction in SCI population have been highlighted in order to advance the clinical knowledge and practices, particularly in the management of persons with SCI. Inference from current state of evidence warrants the assessment of each method during functional task essential for activity of daily living as there is large treatment effect only on isometric contractions. We highlighted the emerging evidence on modulation of FES parameters, electrode positioning to enable asynchronous stimulation, and application of biopotential controlled FES exercising and muscle conditioning through exercise as the major fatigue reducing strategies. Despite the huge opportunities in the substantial independence leading to an improved quality of life for the FES candidates, the level of current evidence shows that the effective fatigue management in FES-induced contractions is still rudimentary. Most studies were conducted within short duration and were too modest, therefore, of limited clinical relevance. Many aspects of the studies were insufficiently researched to draw definitive conclusions. Moreover, several reviewed studies were marked with inadequate randomization, therefore caution should be exercised in interpreting authors’ conclusions. However, the highlighted proof of concepts provide basis for more systematic studies in a wider test population. Although the recent improvement in the commonly used strategies and current interest are of importance, evaluation of the strategies while employing more vigorous methodological design, particularly, in non-isometric contraction settings will be more clinically relevant.

## Supporting Information

S1 File Keywords and search strategies. (DOCX)

S2 File PRISMA 2009 Checklist. (DOCX)
