# Supplementary material for: Strategies for Rapid Muscle Fatigue Reduction during FES Exercise in Individuals with Spinal Cord Injury: A Systematic Review
Source: PLoS One. 2016 Feb 9;11(2):e0149024. doi: 10.1371/journal.pone.0149024 (PMC4747522; doi:10.1371/journal.pone.0149024)
Supplement: S1 File — (DOCX) [file pone.0149024.s001.docx]

MEDLINE search strategy from which potential articles were identified.

(Limited to studies on humans and studies in English language only)

Set A (combined by OR): ‘spinal cord injury’, ‘paralysis’, ‘paraplegia’, ‘tetraplegia’.

Set B (combined by AND): ‘Set A’, ‘muscle fatigue reduction’, muscle fatigue delay”.

Set C (combined by AND): ‘Set B’, ‘functional electrical stimulation’, OR ‘electrical stimulation’.

Set D (combined by AND): ‘Set C’, ‘therapy’, OR ‘contractions’, OR ‘walking’, OR ‘stepping’, OR ‘standing’ OR ‘cycling’.
